# Staphylococcal Enterotoxins and Toxic Shock Syndrome Toxin-1 and Their Association among Bacteremic and Infective Endocarditis Patients in Egypt

**DOI:** 10.1155/2020/6981095

**Published:** 2020-12-18

**Authors:** Heba M. Elsherif, Zeinab H. Helal, Mona R. El-Ansary, Zeinab A. Fahmy, Wafaa N. Eltayeb, Sahar Radwan, Khaled M. Aboshanab

**Affiliations:** ^1^Department of Microbiology, Faculty of Pharmacy, Misr International University (MIU), PO 19648 Cairo, Egypt; ^2^Department of Microbiology, Faculty of Pharmacy, Al-Azhar University, PO 11651 Cairo, Egypt; ^3^Department of Biochemistry, Modern University for Technology and Information (MTI), PO 11566 Cairo, Egypt; ^4^Department of Cardiology, Faculty of Medicine, Ain Shams University (ASU), PO 11566 Cairo, Egypt; ^5^Department of Microbiology & Immunology, Faculty of Pharmacy, Ain Shams University (ASU), PO 11566 Cairo, Egypt

## Abstract

**Purpose:**

Infective endocarditis (IE) is a major complication in patients with bacteremia of *Staphylococcus* (*S.*) *aureus* infection. Our aim was to determine the association of the major Staphylococcal superantigens (SAgs), including Staphylococcal enterotoxins (SEs) and toxic shock syndrome toxin-1 (TSST-1), among hospitalized patients diagnosed with bacteremia and those with IE.

**Methods:**

This study was conducted on 88 patients; of these, 84 (95.5%) had two positive blood cultures. Eighteen out of the 84 patients (21.4%) were diagnosed based on the modified Duke criteria by a cardiologist to have IE. The recovered isolates were screened phenotypically using ELISA followed by molecular analysis of *sea*, *seb*, *sec*, *sed*, *see*, and *tsst-1*, the major SAg coding genes, and the obtained findings were statistically analyzed.

**Results:**

Phenotypic screening for SE production of 26 selected Staphylococci (15 isolated from the IE patients (10 *S. aureus* and 5 coagulase negative staphylococci (CoNS)) and 11 from bacteremic patients (10 *S. aureus* and 1 CoNS)) using ELISA revealed that 12/26 (46%) isolates were SE producers. PCR analysis showed that 19 (73%) isolates were PCR positive for SAg genes with the highest prevalence of the *sea* gene (79%), followed by *seb* (63%) and *tsst-1* (21%). The least frequent gene was *sed* (5.3%). Statistical correlations between bacteremic and IE isolates with respect to prevalence of SAgs showed no significant difference (*P* value = 0.139, effect size = 0.572) indicating no specific association between any of the detected SAgs and IE.

**Conclusion:**

There is high prevalence of SEs among clinical isolates of Staphylococci recovered from patients suffering bacteremia and those with IE. No significant difference was found among Staphylococcal isolates recovered from patients with bacteremia or IE regarding both phenotypic and genotypic detection of the tested SAgs.

## 1. Introduction

*S. aureus* is a dangerous and versatile human pathogen because of its ability to cause various types of infections, including skin and soft tissue infections, pneumonia, bloodstream infections (BSIs), osteomyelitis, and infective endocarditis (IE) [1, 2]. A higher mortality rate (15–25%) was recently reported from serious *S. aureus* infections, particularly bacteremia and endocarditis [3, 4]. IE is a major devastating complication of Staphylococcal bacteremia [4, 5]. In 2018, Asgeirsson et al. reported that *S. aureus* is the foremost cause of IE where it comprises about 15–40% of all IE cases worldwide [4]. IE is primarily caused by bacteria or fungi that may progress to serious cardiac complications [6]. IE is typically characterized by the development of “cauliflower-like” vegetations, consisting of host factors including fibrin, platelets, and bacterial aggregates on the damaged endothelium of heart valves [7, 8]. Patient groups at high risk of developing *S. aureus* bacteremia include patients with higher prevalence of colonization, immunocompromised patients, and patients on hemodialysis. Also, insertion of prosthetic devices also increases the risk of infection [9, 10]. However, the involvement of bacterial complications still needs further studies to be identified as the basis of Staphylococcal virulence, and switching between commensal and pathogenic phenotypes is still unclear. Based on the European guidelines, clinical picture, microbiological analysis, and echocardiographic investigations are the most helpful techniques that are usually used for the diagnosis of IE [11]. The widely accepted Duke criteria provide high sensitivity and specificity for the diagnosis of IE where a series of major and minor clinical and pathologic criteria are implemented [12, 13].

Superantigens (SAgs) are significant virulence factors that contributed to a variety of pathological conditions, including pneumonia, soft tissue infections, toxic shock syndrome, and IE. They have been found to play a very critical part in the pathogenesis of IE [7, 8]. It was recently reported that SAgs particularly the *S. aureus* enterotoxins play important role in the induction of asthma of hospitalized patients by inducing IgE production [14]. *S. aureus* strains secrete up to 23 of at least 24 serologically distinct SAgs including TSST-1, SEs, and the SE-like (SE-l) [9, 15, 16]. These SAgs have the distinctive capability to concurrently bind both major histocompatability complex and the T-cell receptor, exerting immune response 20% greater than that of ordinary antigens [17, 18]. This immune response is associated with a substantial discharge of various inflammatory cytokines and several interleukins which could have a direct cytotoxic effect on the endothelial cells [8]. Because of the presence of the adhesion surface molecules located on the *S. aureus*, it becomes able to adhere to the cardiac endothelial cells and directly release the cytokines, and therefore, the inflammation of the endocardium is initiated causing IE [19]. Despite the advancements in the field of therapies and infection control, both the morbidity and mortality rates associated with IE have not declined [8, 13]. However, the situation of IE becomes worse upon the emergence of multidrug-resistant strains such as methicillin-resistant *S. aureus* (MRSA) [3, 20]. According to literature, the prevalence of SEs and TSST-1 and their production among *Staphylococcus* clinical isolates associated with IE, particularly in Egypt, have not been well studied. Accordingly, we assessed the prevalence of the SEs and TSST-1 and studied their associations among patients diagnosed with bacteremia and IE.

## 2. Methods

### 2.1. Clinical Specimens and Patient Data

This retrospective study was conducted in El-Demerdash Hospital Ain Shams University and Ain Shams University Specialized Hospital, Cairo, Egypt. Two Blood specimens were collected from each of the 88 bacteremic patients during the period from November 2015 to February 2017. A total of 84 (95.5%) blood specimens showed positive blood culture; of these, 18 (21.4%) specimens were collected from patients diagnosed by the cardiologist to have IE based on the modified Duke criteria [21–23]. Information for these patients has been obtained from their medical records in the hospital. Blood specimens were collected from patients having fever and preferably on early admission. Patients who did not meet the inclusion criteria such as those without fever or those who have been admitted to the hospital for more than one week were excluded from this study. This could be due to the high probability of finding their blood cultures negative, especially if they have already started antibiotic treatment for several days. The whole study was approved by the Faculty of Pharmacy, Ain Shams University Research Ethics Committee (ENREC-ASU-Nr. 65) where both informed and written consent were obtained from patients or parents of patients after explaining the study purpose.

### 2.2. Identification and Antimicrobial Susceptibility Testing for the *Staphylococcus* Isolates

Identification of Gram-positive and Gram-negative isolates was performed according to Bergy's Manual using the standard identification procedures [24]. All Staphylococcal isolates were subjected to susceptibility testing against vancomycin (30 *μ*g), clindamycin (2 *μ*g), gentamicin (10 *μ*g), and ciprofloxacin (5 *μ*g) using the modified Kirby Bauer disk diffusion method as recommended by CLSI 2010 and 2016 guidelines [25, 26]. Phenotypically, MRSA isolates were identified by their resistance to the cefoxitin disk (30 *μ*g) as recommended by CLSI, 2016 [25]. *S. aureus* ATCC 25923 standard strain was used for the quality control of antimicrobial susceptibility tests.

### 2.3. Phenotypic Detection of SEs Using ELISA

The presence of SE types A, B, C, D, and E in bacterial supernatants was assessed using a VIDAS® Staph enterotoxin II kit (BioMerieux, France), following the manufacturer's protocol. A result with a test value that is less than the threshold value (<0.13) indicated that the sample either does not contain SE or the toxin concentration was below the detection limit. On the other hand, a result with a test value that is ≥0.13 indicated the presence of any type of enterotoxins.

### 2.4. Molecular Analysis of SEs and TSST-1

Genomic DNA purification was carried out using the Thermo Scientific GeneJET Genomic DNA Purification Kit (Thermo Scientific, UK), following the manufacturer's protocol. As shown in Table 1, six pairs of primers were used for the PCR amplification of the *sea*, *seb*, *sec*, *sed*, *see*, and *tsst* genes, coded for SE types A, B, C1, D, E, and TSST-1, respectively [27]. Each PCR reaction contained 12.5 *μ*l of Dream*Taq* Green PCR Master Mix (2X), 100 pmol/*μ*l of each primer for each gene, 100 nmole of chromosomal DNA, and continued up to 25 *μ*l with sterile nuclease-free water. DNA amplification was performed using a Horizontal Thermocycler (Biometra, Germany), with the following thermal cycling profile: initial denaturation step at 94°C for 5 min, followed by 35 cycles of denaturation at 95°C for 2 min, annealing at 50°C for 2 min, and extension at 72°C for 1 min, followed by final extension at 72°C for 7 min. The PCR products were analyzed using 2% agarose gel electrophoresis and verified by DNA sequencing [28].

### 2.5. Statistical Analysis

Statistical analysis was performed using Minitab software, version 18.1. Fisher's exact test was used for comparisons related to qualitative data. ELISA and the total number of detected gene data showed nonparametric distribution, so Mann-Whitney *U* was used for the comparisons. A significance level of 0.05 was used.

## 3. Results

### 3.1. Study Population

The study was conducted on 84 bacteremic patients having two positive blood cultures with the same isolated microorganism. It included 65 male (77.3%) and 19 female (22.6%) patients. Based on the modified Duke criteria, 18/84 (21.4%) patients were diagnosed by the cardiologist with IE. A summary of these 18 patients' demographics and clinical characteristics is presented in Table 2. The eighteen patients showed native valve endocarditis where 14 (77.7%) patients had damage in one single valve and 4 (22.2%) patients had defects in two valves. Among these patients, the tricuspid valve was the most commonly affected (9; 50%) followed by the mitral (8; 44.4%) and then aortic (5; 27.7%).

### 3.2. Microbial Population and Antimicrobial Susceptibility

For the laboratory examination of 84 positive culture specimens, a total of 85 clinical isolates (83 specimens gave single and 1 specimen was double culture) were recovered; of these, 59 (69.4%), 22 (25.8%), and 4 (4.7%) were Gram-positive, Gram-negative, and *Candida* spp., respectively. Among the Gram-positive isolates, the most common organisms identified were the CoNS (28; 47.5%), followed by *S. aureus* (26; 44.1%), *Streptococcus* spp. (3; 5.1%), and *S. intermedius* (2; 3.4%). The most common CoNS isolated were *S. epidermidis* representing 53.5%, followed by the *S. lugdunensis* and *S. haemolyticus* representing 25% and 22.4%, respectively.

As shown in Table 3, among the *S. aureus* isolates, 24 (92.3%) were MRSA. The susceptibility of *S. aureus* isolates against vancomycin, clindamycin, ciprofloxacin, and gentamicin were 92.3%, 65.4%, 61.5%, and 50%, respectively. On the other hand, the susceptibility of CoNS against vancomycin, ciprofloxacin, gentamicin, clindamycin, and cefoxitin was 96.4%, 35.7%, 32.1%, 32.1%, and 3.5%, respectively.

### 3.3. Microbiology of the IE Cases

Out of the 18 blood specimens collected from IE patients, 19 microbial isolates were recovered. The most common pathogen was *S. aureus* (10; 52.6%), followed by CoNS (5; 26.3%) and Gram-negative isolates (3; 15.7%) while one isolate was from the *Candida* spp. (5%).

### 3.4. Phenotypic SE Detection

Twenty-six Staphylococcal isolates were selected for the detection of SEs using ELISA. The priority in the selection was for the 15 Staphylococci isolated from the IE patients (10 *S. aureus* and 5 CoNS). Then, we selected 11 of the Staphylococci isolated from bacteremic patients (10 *S. aureus* and 1 CoNS). Only, 12 isolates (46%) were positive SE producers while the remaining 14 isolates (54%) were negative. The 12 positive SE isolates were 9 (75%) from IE and 3 (25%) from bacteremic patients without IE. The mean for the ELISA score was 0.995, and the median was 0.05. The P50 was 0.05, which indicated that 50% of the tested isolates showed an ELISA score less than 0.05. The value of P75 was 2.04 which indicated that 75% of the isolates showed an ELISA score less than 2.04, while the P90 was 2.108 which means that 90% of the isolates revealed a score less than 2.108.

### 3.5. Molecular Analysis of SEs and TSST-1

**Figure 1:**
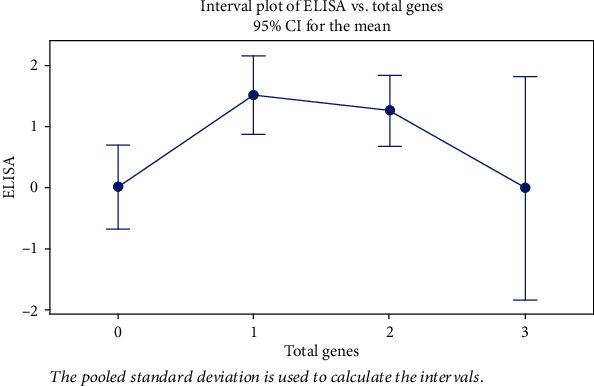
Interval plot of ELISA versus total genes.

As shown in Table 4, out of the 26 isolates, 19 (73%) harbored at least one SAg gene; of these, 8 isolates were positive for only one and 11 were positive for two or more genes (Figures 1S, 2S, and 3S). Out of the 19 positive isolates, 14 (73.7%) and 5 (26.3%) were from IE and bacteremic patients without IE, respectively. The most frequent gene found among the tested isolates was *sea* gene representing 79% of the isolates, followed by the *seb* and the *tsst-1* genes representing 63% and 21%, respectively. The least frequent gene was *sed* representing only 5.3%. However, *sec* and *see* genes were absent in any of the tested isolates. There was a statistically significant difference in the overall prevalence of the *sea* gene compared to the other genes detected among the tested isolates (*P* = 0.001). To compare between *S. aureus* and CoNS genetic profile, among *S. aureus* isolates, the *sea* gene was the most prominent gene and was identified in 65% of the studied *S. aureus* isolates; however, it was only identified in 33.3% of CoNS. On the other hand, *seb*, *tsst*, and *sed* genes were present in 50%, 20%, and 5% of *S. aureus* isolates, respectively. Among CoNS, *seb* and *tsst* genes were both present each in 33.3%, while the *sed* gene was not detected in any of the tested isolates.

### 3.6. Correlation between Phenotypic and Genotypic Detections of SAg among Staphylococci

We studied the statistical correlations between IE and bacteremic isolates with respect to the phenotypic and genotypic detection of SAg. The Mann-Whitney Rank Sum Test and Fisher's exact test were performed to test the significance of the SAg as detected phenotypically using the ELISA test and genotypically using PCR amplification among bacteremic and IE *Staphylococcus* isolates. As shown in Table 5, no significant difference has been found when comparing the results of the ELISA test among IE and bacteremic isolates (*P* value = 0.085, effect size = 0.677). There was no statistically significant difference between prevalence of *sea*, *seb*, *sed*, and *tsst-1* genes among bacteremic and IE patients (*P* value = 0.426, effect size = 0.212; *P* value = 0.453, effect size = 0.168; *P* value = 0.423, effect size = 0.234; and *P* value = 0.113, effect size = 0.365, respectively). There was also no statistically significant difference between the total number of SAg genes present among IE and bacteremic Staphylococcal isolates (*P* value = 0.139, effect size = 0.572).

We studied the correlation between the ELISA results among Staphylococcus isolates in association with the respective genes detected. As shown in Table 6, isolates with the *sea* gene showed statistically significantly higher median ELISA results than isolates without the *sea* gene (*P* value = 0.005, effect size = 1.215). There was no statistically significant difference between ELISA results in isolates with and without *seb* genes (*P* value = 0.978, effect size = 0.010). As regards the *sed* gene, no statistical comparison was performed because there was only one isolate harboring this gene. As presented in Table 7 and Figure 1, pairwise comparison using analysis of variance among different groups showed a significant difference between isolates with no genes against isolates with 1 or 2 genes with respect to the average ELISA score (*P* = 0.012), while no significant differences were detected between isolates within the same group having either 1, 2, or 3 genes.

## 4. Discussion

Globally, BSIs are the major cause of infectious disease morbidity and mortality [29, 30]. Recently, the epidemiology of BSIs has been changed, as a result of many factors, for example, increasing globalization, emerging antimicrobial-resistant organisms, changing population demographics, and modifications in health care delivery models [29]. It is reported that *S. aureus* is the second most common species causing BSIs [30]. Genetic variation of genes that encode for SAg production by *Staphylococcus* spp. may contribute to the occurrence of IE in the course of bacteremia. Therefore, it is important to highlight the prevalence of SAgs in IE as well as in bacteremic patients.

The cornerstones of clinical diagnosis of IE rely on integration of clinical, microbiological, echocardiography, and laboratory findings; these are underlined in the modified Duke criteria for the diagnosis of IE. The use of the Duke criteria is highly recommended in the guidelines thus allowing correct diagnosis and rapid treatment [31]. In the present study, the mean age of our patients was 33 ± 11.3 years. The majority of patients were male representing 83%, and the remaining 17% were female patients. Similar findings were previously reported [6, 13, 32]. The higher incidence of the young, male IE patients in our study can be correlated to the high number of injection drug users (IDUs), which is considered a problem related mainly to the young males in our Egyptian society [33]. The study revealed that 75% of the IDUs were HCV positive [33]. These results were in agreement with those reported in a previous study [32]. Our findings were in accordance with results reported by a study conducted by Ghosh et al., in which 91% of the patients had fever as the most prevalent symptom [34]. All patients included in the study did not have underlying preexisting valvular diseases, since the majority of our IE patients were IDUs not cardiac patients. Therefore, it was common to find that patients who had native valve IE as the estimated prevalence of IDUs have increased worldwide [32–34]. Consequently, the number of IE cases linked to IDUs has increased as well. A recent study reported that the number of patients diagnosed with IE has increased dramatically over the last decade in a way that reflects the increase in the number of IDUs [35]. Moreover, patients with postoperative prosthetic valves follow a strict follow-up with adequate medical care, so the prevalence of IE among these patients decreased significantly [34]. Among the IDUs, the tricuspid valve was the most commonly affected valve (66.7%). A similar finding was observed in a previous study [36].

The results of our study revealed that the higher prevalence was for the Gram-positive isolates (69.4%) causing BSIs compared to the Gram-negative isolates (25.8%). Among the recovered Gram-positive isolates, the highest prevalence was for the CoNS (50.8%), followed by *S. aureus* (44.1%), and the least prevalence was for the *Streptococcus* spp. (5%). The epidemiology of BSIs towards Gram-positive pathogens could be due to the increase in risk factors in the populations including older age, diabetes, end-stage renal disease, intracardiac devices, increased use of invasive procedures, and IV drug use as well as frequent insertion of central venous catheters. All these mentioned patient risk factors may lead to the development of complicated BSIs with MRSA as well as CoNS [37, 38].

When assessing the susceptibility of *S. aureus* to different antimicrobial agents, our results revealed that MRSA was responsible for most *S. aureus* bacteremia (92.3%), and also, cefoxitin resistance among CoNS was highly noticed with a percentage of 93.3%. Our results were in agreement with the results of several studies conducted in Egypt reporting the high frequency of MRSA among *S. aureus* isolates with percentages of 40% and 88%, respectively [39, 40]. As for the susceptibility of the isolates to vancomycin, 92.3% of the *S. aureus* isolates were susceptible, and 93.3% of the CoNS were also susceptible to vancomycin. Therefore, vancomycin remains the drug of choice and the most appropriate and commonly used treatment for Staphylococcal BSIs. In particular, vancomycin is endorsed by the Infectious Diseases Society of America (IDSA) MRSA guidelines as the main treatment choice for MRSA bacteremia [41], although in 2016 the CLSI recommended MIC tests to be performed to determine the susceptibility of Staphylococci to vancomycin, as the disk test does not differentiate vancomycin-intermediate isolates of *S. aureus* from vancomycin-resistant strains. In our study, we only performed the disk test as we found that 92.3% of the *S. aureus* isolates and 96.4% of the CoNS isolates were sensitive to vancomycin. It was not essential to compare between the intermediate and the resistant isolates especially that most of them did not show any zone of inhibition; therefore, they were considered resistant.

IE usually results from infection by Gram-positive bacteria and infrequently from Gram-negative bacteria. This may be due to the fact that the Gram-positive bacteria have the capability to adhere and inhabit damaged valves [42]. In addition, Gram-positive bacteria are armed with numerous superficial adhesins that arbitrate attachment to extracellular host matrix proteins [43]. *S. aureus*, *Streptococcus* spp., and enterococci are the most common IE pathogens which are responsible for more than 80% of IE cases [44, 45]. Historically, *Streptococcus* species have been the main causative microorganisms of IE. However, other pathogens have gained importance. *S. aureus* has become the predominant causative organism in the world, in both hospital settings and the community, followed by CoNS [1, 39]. Therefore, *S. aureus* was the most commonly isolated pathogen (52.6%), followed by CoNS (26.3%). The same finding was reported in a study conducted by Fatima et al., where *S. aureus* was found to be the predominant organism causing IE (38%) [38]. For the CoNS, it was traditionally known to be a rare cause of native valve IE [38]. However, the rates of CoNS bacteremia and CoNS IE had increased in the past years [38]. Several investigations have described the emerging importance of CoNS to cause native valve endocarditis in both community and healthcare settings with a high potential to cause complications and death [46, 47].

Concerning the epidemiology of SAgs, to date, several SAgs have been identified, and globally, SAg genes have been found in over 70% of *S. aureus* isolates [48, 49]. Various immunological and molecular methods have been developed for the phenotypic and genotypic detection of SAgs. The prevalence of five SE encoding genes (A-E) as well as the *tsst-1* was investigated by PCR amplification. The results revealed that among the total Staphylococcal isolates, 57.9% carried two or more genes of the assessed SAgs, while 42% of the isolates had only one gene. From the 15 isolates recovered from IE patients, 93% of the isolates had at least one SAg gene and 53% had two SAg genes. In addition, among *S. aureus* isolates, 80% of the isolates had at least one SAg gene, and these were in accordance with those reported in several studies, where they found that 70-90% of the isolates had one SAg gene [48, 50]. However, a lower percentage was reported by Chung et al., where out of the 124 isolates, 63 *S. aureus* isolates (50.8%) had at least one SAg gene [51]. Among the *S. aureus* isolates of SAg genes, *sea* was the most commonly found gene followed by the *seb* gene and *tsst*-*1*, and the least prevalence was for the *sed*. The genes coding for enterotoxins C1 and E were not found among the tested isolates. Our results were in accordance with other reports [52, 53]. They found the highest frequency for the *sea* gene, followed by the *seb* gene and *sed* genes. In contrast to our study, Nhan et al. found that *sec* and *seb* genes were the most prevalent toxin genes in their study [54]. The molecular detection of SAg was found to be more sensitive and efficient than the ELISA test, since the results of the PCR amplification revealed that 20 out of the 26 isolates were positive for SAg genes. However, only 12 isolates were positive for enterotoxin production. This could be due to the low-level production of enterotoxins by some isolates, which are not detected by VIDAS ELISA.

In spite of this, the question remains as to whether IE *Staphylococcus* isolates differ from non-IE bacteremia isolates. Our results showed no significant difference between Staphylococcal IE and bacteremia isolates with respect to both phenotypic and genotypic detection of the most commonly found SAgs. Our data rule out the possibility of a single specific SAg responsible for the occurrence of IE in the course of Staphylococcal bacteremia. Our results were in accordance with Bouchiat et al. and Gallardo-García et al., where they found no association between any SAg and IE [52, 55]. On the other hand, in 2014, Chung et al. analyzed a series of 124 *S. aureus* isolates in IE and found a significant correlation between SAgs and IE [51]. Moreover, in 2012, Tristan et al. found that the genes encoding toxic shock syndrome toxin-1 and Staphylococcal enterotoxin A, the two major SAgs from *S. aureus*, were enormously widespread in IE isolates from the USA, 93.9% and 64.9%, respectively [50]. Accordingly, they suggested that IE isolates carry specific virulence factors that differ from those found in isolates tested from patients suffering other infections [50].

## 5. Conclusion

In conclusion, there was a statistically significant difference between phenotypic and the genotypic detection methods among the Staphylococcal tested isolates. On the other hand, our study revealed that no significant difference has been found between Staphylococcal IE and bacteremia isolates regarding both phenotypic and genotypic detection of the most common SAgs. Accordingly, all patients with bacteremia of Staphylococcal origin are suspected for IE and need a follow-up to confirm that bacteremia has not been complicated with IE.

It is important to note that one limitation of the study was the inability to establish SAg gene expression *in vitro*. However, detection of SAg gene expression will be made in our future research.

## Figures and Tables

**Table 1:** Primer sequences and expected sizes of PCR products.

Gene	Primer sequence (5′-3′)	Expected size (bp)	Tm	Reference
*Sea* (Staphylococcal type A enterotoxin)	F-GGTTATCAATGTGCGGGTGG R-CGGCACTTTTTTCTCTTCGG	102	50	[26]
*Seb* (Staphylococcal type B enterotoxin)	F-GTATGGTGGTGTAACTGAGC R-CCAAATAGTGACGAGTTAGG	164	50	[26]
*Sec* (Staphylococcal type C1 enterotoxin)	F-AGATGAAGTAGTTGATGTGTATGG R-CACACTTTTAGAATCAACCG	451	50	[26]
*Sed* (Staphylococcal type D enterotoxin)	F-CCAATAATAGGAGAAAATAAAAG R-ATTGGTATTTTTTTCGTTC	278	50	[26]
*See* (Staphylococcal type E enterotoxin)	F-AGGTTTTTTCACAGGTCATCC R-CTTTTTTTTTCTTCGGTCAATC	209	50	[26]
*tsst-1* (toxic shock syndrome toxin-1)	F-ACCCCTGTTCCCTTATCATC R-TTTTCAGTATTTGTAACGCC	326	50	[26]

**Table 2:** Demographics and clinical characteristics of 18 patients with IE.

Variable	Number	Percentage (%)
Gender		
Male	15	83%
Female	3	17%
Valve type		
(i) Native	18	100%
(a) Tricuspid (T)	9	50.0%
(b) Mitral (M)	8	44.5%
(c) Aortic (A)	5	27.7%
(ii) Prosthetic	0	0%
Number of valve affected		
(a) Single valve	14 (77.7%)	
(b) Two valves	4 (22. 2%): T+M (3; 75%) T+A (1; 25%)	
Age (years)	17-48	
Smoker		
Yes	14	78%
No	4	22%
Addiction		
Injection drug users (IDUs)	12	66.6%
Hepatitis C virus (HCV)		
Yes	10	56%
No	8	44%
Surgery performed		
Yes	4	22%
No	14	78%
Admitted to ICU		
Yes	2	11%
No	16	89%
Comorbidities		
On hemodialysis	1	5.5%
Diabetes	—	0%
Cancer	1	5.5%
No comorbidities	16	89%
Complications		
Renal failure	1	5.5%
Pneumonia/lung abscess	2	11%
Peripheral septic emboli	1	5.5%
No complications	14	78%

**Table 3:** Antimicrobial susceptibility pattern of the recovered *Staphylococci.*

Antimicrobial agent	Susceptibility pattern of Staphylococci isolates					
	*S. aureus* (*N* = 26)			CoNS (*N* = 28)		
	Sensitive	Intermediate	Resistant	Sensitive	Intermediate	Resistant
	No. (%)	No. (%)	No. (%)	No. (%)	No. (%)	No. (%)
Vancomycin	24 (92.3)	0	2 (7.7)	27 (96.4)	0	1 (3.5)
Clindamycin	17 (65.4)	0	9 (34.6)	9 (32.1)	2 (6.6)	17 (60.7)
Gentamicin	13 (50.0)	1 (4)	12 (46)	9 (32.1)	2 (6.6)	17 (56.6)
Cefoxitin	2 (7.7)	0	24 (92.3)	1 (3.5)	0	27 (96.4)
Ciprofloxacin	16 (61.5)	0	10 (38.4)	10 (35.7)	3 (10)	15 (50)

**Table 4:** Distribution of SAg genes among *Staphylococci.*

Genes	No. (%) of Staphylococcal isolates harbored SAg genes (*N* = 26)	
Detection of gene(s)	Positive: 19 (73%)	IE patients 14 (73.7%); bacteremic patients without IE, 5 (26.3%)
	Negative: 7 (27%)	
One SAg	8 (42%)	*P* value = 0.491
≥2 SAg	11 (57.9%)	
Types of genes detected		
*Sea*	15 (79%)	*P* value = 0.001
*Seb*	12 (63%)	
*Sec*	0 (0%)	
*Sed*	1 (5.3%)	
*See*	0 (0%)	
*tsst-1*	4 (21%)	

**Table 5:** Descriptive statistics, results of Fisher's exact test and Mann-Whitney *U* test for comparison between ELISA results and detected genes among bacteremic and IE isolates.

Outcome	IE (*n* = 15)	Bacteremia (*n* = 11)	*P* value	Effect size
ELISA (median (range))	1.92 (0-2.59)	0 (0-2.05)	0.085	*d* = 0.677
Detected genes (*n* (%))				
Sea	10 (66.7%)	5 (45.5%)	0.426	*v* = 0.212
Seb	8 (53.3%)	4 (36.4%)	0.453	*v* = 0.168
Sed	0 (0%)	1 (9.1%)	0.423	*v* = 0.234
tsst1	4 (26.7%)	0 (0%)	0.113	*v* = 0.365
Total number of genes (median (range))	2 (0-2)	1 (0-3)	0.139	*d* = 0.572

^∗^Significant at *P* ≤ 0.05.

**Table 6:** Median, range, and results of Mann-Whitney *U* test for comparison between ELISA results in association with the detected genes.

Gene	Present	Absent	*P* value	Effect size (*d*)
*sea*	2.03 (0-2.59)	0 (0-2.03)	0.005^∗^	1.215
*seb*	0.01 (0-2.59)	0.95 (0-2.08)	0.978	0.010
*sed*	Only one case	0.05 (0-2.59)	Not computed	

^∗^Significant at *P* ≤ 0.05.

**Table 7:** Grouping information using the Tukey method and 95% confidence.

Total genes	Number	Mean	Grouping
1	8	1.519	A
2	10	1.268	A
0	7	0.0071	B
3	1	0.0000	A

Group A: 1, 2, or 3 genes present. Group B: no genes.

## Data Availability

All data generated or analyzed during this study are included in this manuscript.

## Supplementary Materials

Supplementary Materials Figure 1S: Agarose gel electrophoresis pattern showing PCR-amplified products in multiplex PCR for Staphylococcal *sea, sec* and *see* genes. Lanes 1 and 8: 1 Kb molecular weight marker or ladder (Thermo Fischer Scientific, UK).Lanes 3, 5 and 6: Samples loaded in these wells showed a PCR product of the amplified gene *sea* ≈ 102bp. Figure 2S: Agarose gel electrophoresis pattern showing PCR-amplified products in multiplex PCR for Staphylococcal *seb* and *sed* genes. Lanes 1 and 8: 1 Kb molecular weight marker or ladder (Thermo Fischer Scientific, UK).Lanes 3, 5 and 6: Samples loaded in these wells showed a PCR product of the amplified gene *seb* ≈ 164bp. Lane 3: Sample loaded in the well is showing a PCR product of the amplified gene *sed* ≈ 278bp. Figure 3S: Agarose gel electrophoresis pattern showing PCR-amplified products in uniplex PCR for Staphylococcal *tsst-*1 gene.Lanes 1 and 8: 1 Kb molecular weight marker or ladder (Thermo Fischer Scientific, UK).Lanes 2 and 5: Samples loaded in these wells showed a PCR product of the amplified gene *tsst-1* ≈ 326 bp.
