# Supplementary material for: Staphylococcal Enterotoxins and Toxic Shock Syndrome Toxin-1 and Their Association among Bacteremic and Infective Endocarditis Patients in Egypt
Source: Biomed Res Int. 2020 Dec 18;2020:6981095. doi: 10.1155/2020/6981095 (PMC7762650; doi:10.1155/2020/6981095)
Supplement: Supplementary Materials — Figure 1S: Agarose gel electrophoresis pattern showing PCR-amplified products in multiplex PCR for Staphylococcal sea, sec and see genes. Lanes 1 and 8: 1 Kb molecular weight marker or ladder (Thermo Fischer Scientific, UK).Lanes 3, 5 and 6: Samples loaded in these wells showed a PCR product of the amplified gene sea ≈ 102bp. Figure 2S: Agarose gel electrophoresis pattern showing PCR-amplified products in multiplex PCR for Staphylococcal seb and sed genes. Lanes 1 and 8: 1 Kb molecular weight marker or ladder (Thermo Fischer Scientific, UK).Lanes 3, 5 and 6: Samples loaded in these wells showed a PCR product of the amplified gene seb ≈ 164bp. Lane 3: Sample loaded in the well is showing a PCR product of the amplified gene sed ≈ 278bp. Figure 3S: Agarose gel electrophoresis pattern showing PCR-amplified products in uniplex PCR for Staphylococcal tsst-1 gene.Lanes 1 and 8: 1 Kb molecular weight marker or ladder (Thermo Fischer Scientific, UK).Lanes 2 and 5: Samples loaded in these wells showed a PCR product of the amplified gene tsst-1 ≈ 326 bp. [file 6981095.f1.docx]

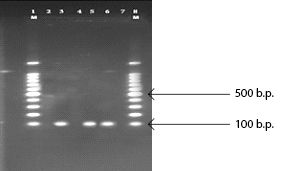


**Figure 1S: Agarose gel electrophoresis pattern showing PCR-amplified products in multiplex PCR for Staphylococcal *sea, sec* and *see* genes.** Lanes 1 and 8: 1 Kb molecular weight marker or ladder (Thermo Fischer Scientific, UK).Lanes 3, 5 and 6: Samples loaded in these wells showed a PCR product of the amplified gene *sea* ≈ 102bp.


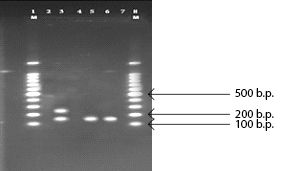


**Figure 2S: Agarose gel electrophoresis pattern showing PCR-amplified products in multiplex PCR for Staphylococcal *seb* and *sed* genes.** Lanes 1 and 8: 1 Kb molecular weight marker or ladder (Thermo Fischer Scientific, UK).Lanes 3, 5 and 6: Samples loaded in these wells showed a PCR product of the amplified gene *seb* ≈ 164bp. Lane 3: Sample loaded in the well is showing a PCR product of the amplified gene *sed* ≈ 278bp.


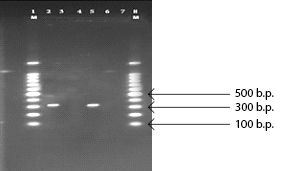


**Figure 3S: Agarose gel electrophoresis pattern showing PCR-amplified products in uniplex PCR for Staphylococcal *tsst-*1 gene.**Lanes 1 and 8: 1 Kb molecular weight marker or ladder (Thermo Fischer Scientific, UK).Lanes 2 and 5: Samples loaded in these wells showed a PCR product of the amplified gene *tsst-1* ≈ 326 bp.
